# Genome-wide survey of putative Serine/Threonine protein kinases in cyanobacteria

**DOI:** 10.1186/1471-2164-8-395

**Published:** 2007-10-30

**Authors:** Xiaowen Zhang, Fangqing Zhao, Xiangyu Guan, Yu Yang, Chengwei Liang, Song Qin

**Affiliations:** 1 Institute of Oceanology, Chinese Academy of Sciences, Nanhai Road, Qingdao, China; 2 Graduate University, Chinese Academy of Sciences, Yuquan Road, Beijing, China; 3 Ocean University of China, Yushan Road, Qingdao, China

## Abstract

**Background:**

Serine/threonine kinases (STKs) have been found in an increasing number of prokaryotes, showing important roles in signal transduction that supplement the well known role of two-component system. Cyanobacteria are photoautotrophic prokaryotes able to grow in a wide range of ecological environments, and their signal transduction systems are important in adaptation to the environment. Sequence information from several cyanobacterial genomes offers a unique opportunity to conduct a comprehensive comparative analysis of this kinase family. In this study, we extracted information regarding Ser/Thr kinases from 21 species of sequenced cyanobacteria and investigated their diversity, conservation, domain structure, and evolution.

**Results:**

286 putative STK homologues were identified. STKs are absent in four *Prochlorococcus *strains and one marine *Synechococcus *strain and abundant in filamentous nitrogen-fixing cyanobacteria. Motifs and invariant amino acids typical in eukaryotic STKs were conserved well in these proteins, and six more cyanobacteria- or bacteria-specific conserved residues were found. These STK proteins were classified into three major families according to their domain structures. Fourteen types and a total of 131 additional domains were identified, some of which are reported to participate in the recognition of signals or substrates. Cyanobacterial STKs show rather complicated phylogenetic relationships that correspond poorly with phylogenies based on 16S rRNA and those based on additional domains.

**Conclusion:**

The number of STK genes in different cyanobacteria is the result of the genome size, ecophysiology, and physiological properties of the organism. Similar conserved motifs and amino acids indicate that cyanobacterial STKs make use of a similar catalytic mechanism as eukaryotic STKs. Gene gain-and-loss is significant during STK evolution, along with domain shuffling and insertion. This study has established an overall framework of sequence-structure-function interactions for the STK gene family, which may facilitate further studies of the role of STKs in various organisms.

## Background

Cyanobacteria, dating back 2.5–3.5 billion years and constituting a single but large taxonomic and phylogenetic group within the domain Eubacteria [1], are characterized by their ability to carry out oxygenic photosynthesis. Moreover, fossilized cyanobacteria appear similar in form to extant species [2]. Cyanobacteria have a pronounced variation in genome size from 1.6 Mb to 9.2 Mb and exhibit extraordinary diversity in terms of morphology and cell activity. They also exhibit the widest range of diversity in ecological habitats of all photosynthetic organisms, including environments that are extremely hot, extremely cold, alkaline and acidic, marine, freshwater, saline, terrestrial, and symbiotic [3]. *Prochlorococcus marinus*, which has the smallest genome size and can be divided into two distinct ecotypes (high-light adapted and low-light adapted), is the dominant photosynthetic prokaryote in the open ocean [4]. The diazotrophic filamentous cyanobacteria have the largest genome sizes and include strains isolated from fresh water (*Anabaena *PCC7120), from a plant-cyanobacterial symbionsis (*Nostoc punctiforme *PCC73102), or from tropical and subtropical oceans (*Trichodesmium erythraeum *IMS101). *Crocosphaera*, a novel genus of marine unicellular diazotrophic cyanobacterium, and *Gloeobacter*, a rod-shaped unicellular cyanobacterium isolated from calcareous rocks, have larger genome sizes (6.3 Mb and 4.6 Mb) than other unicellular cyanobacteria.

The diversity of cyanobacteria is also reflected in the complexity of their signal transduction systems. To cope with changing environmental conditions, cyanobacteria have developed a variety of adaptive mechanisms to respond to external or internal changes. Two-component signal transduction systems, characterized by the transfer of phosphate by a sensor kinase from a His residue on the enzyme to an Asp residue on the response regulator, are widely distributed among bacteria [5,6]. One-component systems, defined as proteins that contain known or predicted input and output domains in a single protein molecule but lack histidine kinase and receiver domains, are considered to be the pre-eminent mechanism for signal transduction in bacteria and archaea, except for cyanobacteria [7]. In contrast, the Ser/Thr-specific protein kinases (STKs) serve as the backbone of the eukaryotes transduction network. However, with the first identification of an STK in *Myxococcus xanthus *in 1991 [8], regulatory STKs have been repeatedly identified in prokaryotes. Protein phosphorylation on serine/threonine residues in cyanobacteria was first revealed by radioactive labeling of proteins in 1994 [9]. Numerous bacterial STK genes have since been predicted within genome sequences [10-12], and they have been associated primarily with three different processes, namely regulation of development, stress responses, and pathogenicity.

According to Hanks and Hunter, canonical Ser/Thr protein kinases contain 12 conserved subdomains [13] that fold into a common catalytic core structure, as revealed by the 3-dimensional structures of several protein-serine kinases. These 12 conserved sequence motifs, about 280 amino acids in length, are named as subdomains I-V, VIa, VIb and VII-XI [13,14]. There are three structural subdomains with separate roles: N-terminal nucleotide-binding domains containing subdomains I-IV, C-terminal phosphotransfer and protein-substrate-binding domains containing subdomains VIa-XI, and the intervening linker containing subdomain V [15]. In addition to the conserved catalytic domains, some STKs contain at least one additional domain, such as FHA, WD40, PAS and GAF [16], which may endow STKs with more complicated functions. For example, the FHA domain can participate in a wide range of processes in bacteria, such as intracellular signal transduction, transcription, protein transport, DNA repair, and protein degradation. Moreover, there is a near-perfect correlation between the presence of FHA-containing proteins and Ser/Thr kinases and phosphatases in bacterial genomes [17].

In cyanobacteria, mutation analyses have revealed the functions of some STKs, such as SpkA and SpkB involved in the cellular motility in *Synechocystis *PCC6803 [18,19], and PknD involved in the regulation of nitrogen metabolism in *Anabaena *PCC7120 [20]. A complete signaling pathway involving a bacteria STK remains to be described, but one may anticipate that some STKs are important or even essential in regulating bacterial activities.

As of January 2007, 19 cyanobacterial genomes have been fully sequenced and 6 are in the draft stages. In addition, more than 20 genomes are in the process of being sequenced. The availability of multiple sequenced genomes has been very helpful in phylogenetic and functional studies of cyanobacterial genes, such as those in restriction-modification systems [21] and two-component systems [22]. Comparative genome analysis has been employed in the study on STKs in archaea [15], mycobacteria [23] streptomyces [24] and cyanobacteria [25,26]. Here we present a detailed analysis of the repertoire of STKs in sequenced cyanobacterial genomes. An attempt has been made to identify all STK sequences encoded in the 19 fully sequenced genomes and 2 draft sequences (*Crocosphaera watsonii *WH8501 and *Nostoc punctiforme *PCC73102). An analysis of STK sequences according to their classifications, conservation, domain organization, phylogeny and evolution are presented in this paper, aiming towards a deep understanding of the biological role of STKs in cyanobacteria.

## Results

### Identification of STK proteins

**Figure 1:**
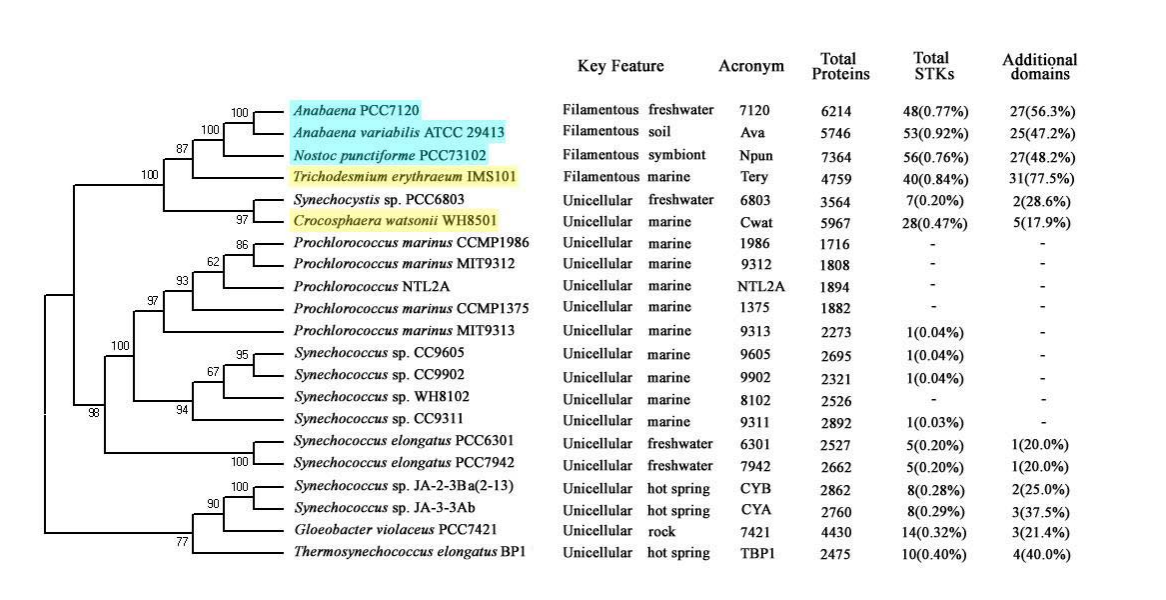
**Phylogenetic tree of the sequenced cyanobacterial strains and STK information**. A phylogenetic tree for 21 sequenced cyanobacteria constructed based on 16s rRNA as was described in Methods. Numbers appearing at the nodes corresponded to the values produced by bootstrap analysis (1000 replicates). Names of marine nitrogen-fixing strains are marked in yellow boxes. Filamentous diazotrophic strains capable of heterocyst differentiation are marked in cyan boxes. This tree is similar to that obtained by Ashby and Houmard [22]. Percentages in brackets represent total STKs as a percentage of total proteins and total additional domains as a percentage of total STKs.

The 21 cyanobacterial genomes fully available from IMG database (Version 2.0, as of Jan 2007) were considered in our analysis (Fig. 1, Table 1). *Synechococcus elongatus *PCC6301 and *Synechococcus elongatus *PCC7942 are virtually identical except for a chromosomal inversion, but both were included in our analysis. Using BlastP and TBlastN programs to look for proteins similar to proven cyanobacterial STKs, we obtained 303 protein sequences from the 21 cyanobacterial genomes, 284 of which were originally annotated as protein kinase or Serine/Threonine Protein Kinase. The remaining 19 proteins were accepted as STKs in this study after sequence alignment and SMART analysis [27]. Seventeen out of these were originally annotated not by their STK domains but by other additional domains, such as ATPase and WD40 repeats, and the last two were annotated as hypothetical proteins [16].

**Table 1:** Cyanobacterial genes encoding Ser/Thr protein kinases

**Gene**^**a**^	**Family**^**b**^	**Additional domains**	**Gene**	**Family**	**Additional domains**
***Prochlorococcus marinus *str. MIT 9313**					
PMT0154	cbSTKI-other				
***Synechococcus *sp. CC9902**					
Syncc9902_0160	cbSTKI-other				
***Synechococcus *sp. CC9605**					
Syncc9605_0116	cbSTKI-other				
***Synechococcus *sp. CC9311**					
sync_0122	cbSTKI-other				
***Synechococcus elongatus *PCC 6301**					
syc0259_c	cbSTKII	Pentapeptide_(2)_	syc0757_d	cbSTKI-other	
syc0428_d	cbSTKI-other		syc0923_d	cbSTKI-other	
syc0438_d	cbSTKI-other				
***Synechococcus elongatus *PCC 7942**					
Synpcc7942_0600	cbSTKI-other		Synpcc7942_1121	cbSTKI-other	
Synpcc7942_0780	cbSTKI-other		Synpcc7942_1294	cbSTKII	Pentapeptide_(2)_
Synpcc7942_1111	cbSTKI-other				
***Synechocystis *sp. PCC6803**					
sll1574(SpkA)	cbSTKI-other		slr0152(SpkG)	cbSTKI-other	
slr1225(SpkF)	cbSTKI-TM		sll0776(SpkD)	cbSTKII	SH3b
slr1443(SpkE)	cbSTKI-other		slr0599(SpkC)	cbSTKI-other	
slr1697(SpkB)	cbSTKII	Pentapeptide_(2)_			
***Synechococcus *sp. JA-2-3Ba(2–13)**					
CYB_0466	cbSTKI-other		CYB_1522	cbSTKII	Pentapeptide
CYB_0522	cbSTKI-other		CYB_1864	cbSTKI-other	
CYB_0637	cbSTKII	GUN4	CYB_2515	cbSTKI-other	
CYB_0949	cbSTKI-other		CYB_2575	cbSTKI-other	
***Synechococcus *sp. JA-3-3Ab**					
CYA_0209	cbSTKI-other		CYA_2262	cbSTKI-other	
CYA_0373	cbSTKI-other		CYA_2333	cbSTKII	TPR_(18)_
CYA_1882	cbSTKII	Pentapeptide	CYA_2756	cbSTKII	GUN4
CYA_2146	cbSTKI-other		CYA_2859	cbSTKI-other	
***Thermosynechococcus elongatus *BP1**					
tlr0445	cbSTKI-other		tlr1460	cbSTKI-other	
tlr1098	cbSTKI-other		tlr1540	cbSTKII	CHASE2
tlr1128	cbSTKII	FHA	tll2222	cbSTKI-other	
tll1205	cbSTKII	CHASE2	tlr2304	cbSTKI-other	
tlr1326	cbSTKII	Pentapeptide_(2)_	tlr2432	cbSTKI-TM	
***Gloeobacter violaceus *PCC 7421**					
gll0054	cbSTKI		glr1346	cbSTKI-other	
glr0422	cbSTKI-other		glr1552	cbSTKI-other	
gll0585	cbSTKII	TPR_(3)_	gll2103	cbSTKI	
glr0657	cbSTKI-other		gll2127	cbSTKI	
glr0665	cbSTKI		glr4017	cbSTKI	
glr0915	cbSTKI-other		glr4072	cbSTKII	TPR_(5)_
glr1096	cbSTKII	TPR_(4)_	glr4107	cbSTKI	
***Crocosphaera watsonii *WH8501**					
CwatDRAFT_0326	cbSTKI-other		CwatDRAFT_3230	cbSTKII	Pentapeptide_(3)_
CwatDRAFT_0327	cbSTKI		CwatDRAFT_3247	cbSTKI	
CwatDRAFT_0457	cbSTKI		CwatDRAFT_3998	cbSTKII	GUN4
CwatDRAFT_0901	cbSTKI		CwatDRAFT_4660	cbSTKI-other	
CwatDRAFT_0902	cbSTKII	ANF	CwatDRAFT_4756	cbSTKI-other	
CwatDRAFT_1649	cbSTKII	PbH1_(3)_	CwatDRAFT_4761	cbSTKI-other	
CwatDRAFT_1757	cbSTKI-other		CwatDRAFT_4762	cbSTKI-other	
CwatDRAFT_1955	cbSTKI-other		CwatDRAFT_4879	cbSTKII	WD40_(7)_
CwatDRAFT_1979	cbSTKI		CwatDRAFT_4783	cbSTKI-other	
CwatDRAFT_2418	cbSTKI		CwatDRAFT_5890	cbSTKI-other	
CwatDRAFT_2794	cbSTKI-other		CwatDRAFT_6040	cbSTKI	
CwatDRAFT_2895	cbSTKI-other		CwatDRAFT_6269	cbSTKI	
CwatDRAFT_3022	cbSTKI		CwatDRAFT_6420	cbSTKI	
CwatDRAFT_3184	cbSTKI-TM		CwatDRAFT_6607	cbSTKI	
***Trichodesmium erythraeum *IMS101**					
Tery_0059	cbSTKII	WD40_(7)_	Tery_2348	cbSTKI-other	
Tery_0088	cbSTKII	FHA	Tery_2556	cbSTKIII	GAF, HiskA
Tery_0184	cbSTKII	WD40_(7)_	Tery_2609	cbSTKII	RDD
Tery_0247	cbSTKII	DUF323	Tery_2829	cbSTKII	DUF323
Tery_0460	cbSTKII	DUF323	Tery_2831	cbSTKII	DUF323
Tery_0461	cbSTKII	DUF323	Tery_2857	cbSTKII	GAF, CYCc
Tery_0462	cbSTKII	DUF323	Tery_3150	cbSTKI	
Tery_0463	cbSTKII	DUF323	Tery_3337	cbSTKII	TPR_(3)_
Tery_0621	cbSTKII	FHA	Tery_3345	cbSTKI-other	
Tery_0858	cbSTKII	TPR_(3)_	Tery_3400	cbSTKII	Pentapeptide_(5)_
Tery_1030	cbSTKII	FHA	Tery_3423	cbSTKI-other	
Tery_1036	cbSTKII	DUF323	Tery_3510	cbSTKI	
Tery_1627	cbSTKII	WD40_(7)_	Tery_3681	cbSTKII	TPR_(7)_
Tery_1668	cbSTKI-other		Tery_3863	cbSTKII	GAF
Tery_1722	cbSTKII	GUN4	Tery_4060	cbSTKII	WD40_(7)_
Tery_1723	cbSTKII	GUN4	Tery_4351	cbSTKII	DUF323_(2)_
Tery_2033	cbSTKII	WD40_(7)_	Tery_4411	cbSTKI-TM	
Tery_2051	cbSTKI-other		Tery_4467	cbSTKII	WD40_(7)_
Tery_2064	cbSTKII	Pentapeptide_(3)_	Tery_4781	cbSTKII	TPR_(11)_
Tery_2107	cbSTKI-other		Tery_4782	cbSTKII	TPR_(10)_
***Anabaena *PCC7120**					
all0192	cbSTKI-other		alr3119	cbSTKII	WD40_(7)_
all0323	cbSTKIII	GAF, HisKA	all3169	cbSTKII	WD40_(7)_
alr0344	cbSTKII	ANF	all3206	cbSTKI-other	
alr0354	cbSTKIII	GAF, HisKA	all3207	cbSTKI-other	
all0438	cbSTKII	WD40_(7)_	alr3268	cbSTKII	Pentapeptide_(2)_
alr0548	cbSTKII	FHA	all3557	cbSTKIII	GAF, HisKA
alr0709	cbSTKIII	GAF, HisKA	all3691	cbSTKIII	GAFGAF, HisKA
alr0710	cbSTKIII	GAF, HisKA	alr3706	cbSTKI-TM	
all0886	cbSTKIII	GAF, HisKA	alr3732(PknE)	cbSTKI-other	
alr0900	cbSTKIII	GAF, HisKA	all3773	cbSTKII	TPR_(10)_
alr1311	cbSTKI		alr3877	cbSTKI-other	
alr1336	cbSTKII	DUF323	alr3997	cbSTKI-other	
all1625	cbSTKIII	GAF, HisKA	alr4141	cbSTKI	
alr1869	cbSTKII	CHASE2	alr4366(PknA)	cbSTKI-other	
all1919	cbSTKI-other		alr4368(PknD)	cbSTKI-other	
alr2258	cbSTKIII	GAF, HisKA	all4518	cbSTKI-other	
alr2259(PknB)	cbSTKI-other		all4668	cbSTKI-other	
all2282	cbSTKIII	GAF, HisKA	all4687	cbSTKIII	GAF, HisKA
all2334	cbSTKI-TM		all4813(PknC)	cbSTKI-other	
alr2411	cbSTKI-other		all4838	cbSTKII	CHASE2
alr2412	cbSTKI-other		alr4949	cbSTKII	GUN4
alr2502	cbSTKII	PbH1_(8)_	alr4954	cbSTKII	FHA
alr2682	cbSTKIII	GAF, PAS, HisKA	all5278	cbSTKI	
all2760	cbSTKI		alr7232	cbSTKII	TPR_(3)_
***Anabaena variabilis *ATCC 29413**					
Ava_0012	cbSTKI		Ava_2851	cbSTKII	WD40_(6)_
Ava_0083	cbSTKIII	GAF, HiskA	Ava_2989	cbSTKI	
Ava_0084	cbSTKI-other		Ava_2990	cbSTKI-TM	
Ava_0153	cbSTKI-TM		Ava_3036	cbSTKI	
Ava_0219	cbSTKI-other		Ava_3308	cbSTKI-other	
Ava_0220	cbSTKI-other		Ava_3310	cbSTKI-other	
Ava_0353	cbSTKII	FHA	Ava_3384	cbSTKI-other	
Ava_0434	cbSTKII	PbH1_(8)_	Ava_3535	cbSTKIII	GAF, HiskA
Ava_0762	cbSTKI		Ava_3584	cbSTKI-TM	
Ava_1153	cbSTKI		Ava_3596	cbSTKIII	GAFGAF, HiskA
Ava_1552	cbSTKII	TPR_(10)_	Ava_3867	cbSTKII	WD40_(7)_
Ava_1592	cbSTKI-other		Ava_3995	cbSTKIII	GAF, HiskA
Ava_1700	cbSTKI-other		Ava_4106	cbSTKI-other	
Ava_1816	cbSTKI-other		Ava_4391	cbSTKI-other	
Ava_1980	cbSTKIII	GAF, HiskA	Ava_4489	cbSTKIII	GAF, HiskA
Ava_1995	cbSTKI-other		Ava_4503	cbSTKIII	GAF, HiskA
Ava_2084	cbSTKI-other		Ava_4504	cbSTKIII	GAF, HiskA
Ava_2109	cbSTKII	CHASE2	Ava_4716	cbSTKIII	GAF, HiskA
Ava_2519	cbSTKI-other		Ava_4764	cbSTKII	CHASE2
Ava_2528	cbSTKI		Ava_4855	cbSTKII	WD40_(7)_
Ava_2684	cbSTKI-other		Ava_4862	cbSTKI-other	
Ava_2791	cbSTKII	ANF	Ava_4863	cbSTKI-other	
Ava_2803	cbSTKIII	GAF, HiskA	Ava_4923	cbSTKII	Pentapeptide_(2)_
Ava_2809	cbSTKI-other		Ava_5054	cbSTKII	DUF323
Ava_2845	cbSTKIII	GAF, PAC, HiskA	Ava_A0032	cbSTKII	TPR_(6)_
Ava_B0009	cbSTKII	TPR	Ava_B0202	cbSTKI-other	
Ava_C0117	cbSTKIII	GAF(PAS, PAC)4PAS HisKA			
***Nostoc punctiforme *PCC73102**					
Npun02000103	cbSTKIII	GAF, HiskA	Npun02004756	cbSTKIII	GAFPASGAF, HiskA
Npun02000163	cbSTKI-other		Npun02004875	cbSTKII	CHASE2
Npun02000400	cbSTKII	WD40_(7)_	Npun02005090	cbSTKI	
Npun02000527	cbSTKI		Npun02005171	cbSTKII	WD40_(7)_
Npun02000941	cbSTKIII	GAF, PAS, PAC, HiskA	Npun02005475	cbSTKI	
Npun02001134	cbSTKIII	GAF, HiskA	Npun02005477	cbSTKII	WD40_(7)_
Npun02001148	cbSTKIII	GAF, HiskA	Npun02005691	cbSTKI-other	
Npun02001995	cbSTKI-other		Npun02006173	cbSTKIII	GAF, HiskA
Npun02002175	cbSTKI		Npun02006290	cbSTKIII	GAF, HiskA
Npun02002176	cbSTKI-other		Npun02006325	cbSTKI-TM	
Npun02002301	cbSTKI-other		Npun02006559	cbSTKI-other	
Npun02002418	cbSTKI-other		Npun02006688	cbSTKI-other	
Npun02002421	cbSTKII	TPR_(10)_	Npun02006812	cbSTKI-TM	
Npun02002449	cbSTKI-other		Npun02006813	cbSTKI	
Npun02002654	cbSTKII	TPR_(10)_	Npun02006829	cbSTKI-other	
Npun02002842	cbSTKI-other		Npun02006849	cbSTKI-other	
Npun02002843	cbSTKI-other		Npun02007024	cbSTKII	WD40_(7)_
Npun02003114	cbSTKI-other		Npun02007058	cbSTKI-other	
Npun02003177	cbSTKII	TPR_(3)_	Npun02007062	cbSTKIII	GAF, HiskA
Npun02003225	cbSTKI-other		Npun02007070	cbSTKII	DUF323
Npun02003227	cbSTKII	WD40_(7)_	Npun02007195	cbSTKII	CHASE2
Npun02003383	cbSTKI-other		Npun02007679	cbSTKIII	GAF, HiskA
Npun02003571	cbSTKII	CHASE2	Npun02007917	cbSTKI-TM	
Npun02003917	cbSTKIII	GAF(PASPAC)2 HiskA	Npun02007926	cbSTKI-TM	
Npun02004022	cbSTKII	TPR_(4)_	Npun02008046	cbSTKI-other	
Npun02004075	cbSTKII	FHA	Npun02008159	cbSTKIII	GAF, HiskA
Npun02004457	cbSTKIII	GAF_(3)_, PAS, PAC, HiskA	Npun02008276	cbSTKI-other	
Npun02004619	cbSTKI		Npun02008678	cbSTKII	Pentapeptide_(2)_

**Figure 2:**
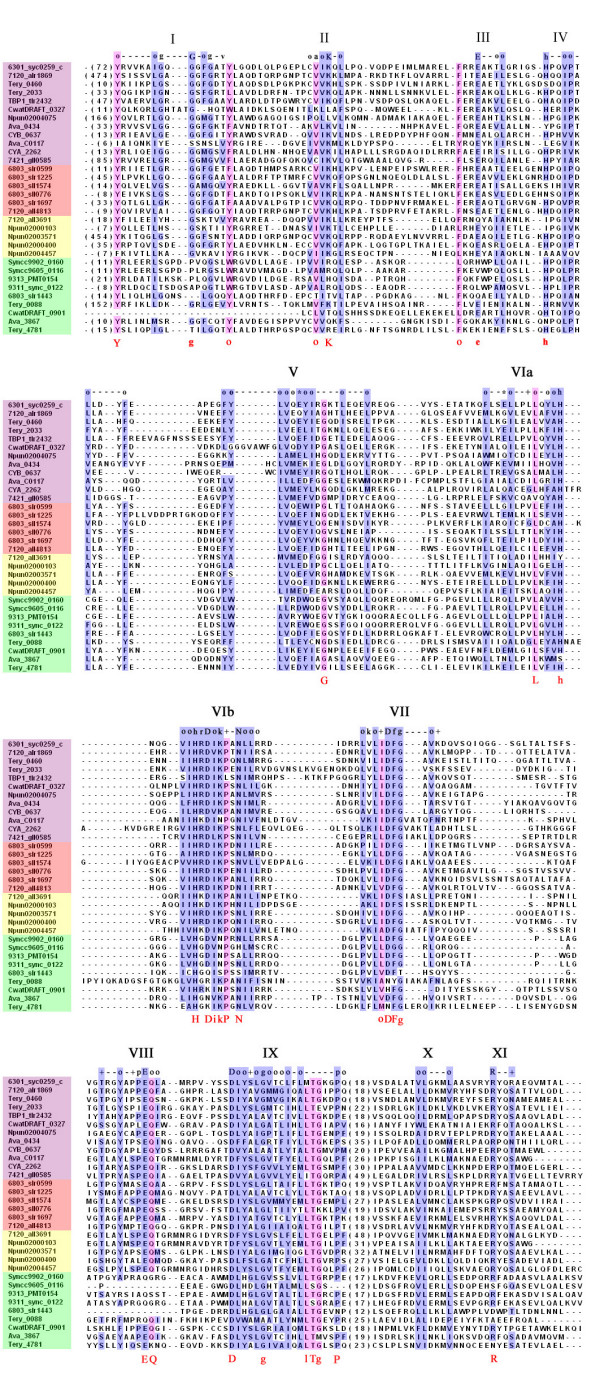
**Conserved domains and sites in cyanobacterial STKs according to Hanks and Hunter**. A total of 32 sequences were used in the alignment of cyanobacterial STKs: 12 sequences in purple were selected to cover the diversity of species and structural characteristics; 6 sequences in red are of proteins reported to possess phosphorylation or autophosphorylation activity; 5 sequences in yellow were not originally annotated as protein kinases or Serine/Threonine Protein Kinases; and 9 sequences in green were those showing some deviations from the canonical domains of Hanks and Hunter [13]. Each sequence is denoted by the species name followed by the gene names. The 12 conserved subdomains are indicated by Roman numerals according to Hanks and Hunter. Highly conserved eukaryotic and cyanobacteria-specific amino-acid residues are indicated above and below the alignment sequences respectively, using the single-letter amino-acid classes of Hanks and Hunter. Symbols, used to denote the various features according to Hanks and Hunter, are as follows: uppercase letters, universally conserved amino acid residues; lowercase letters, highly conserved amino acid residues; o, positions conserving non-polar residues; *, positions conserving polar residues; +, positions conserving small residues with near neutral polarity; and -, positions within a subdomain with no pattern of conserved amino acids. The number in parentheses preceding each sequence refers to the number of amino acid residues preceeding the sequence shown.

17 proteins, all originally annotated as STKs, from the 303 proteins identified by sequence similarity lack all or part of at least one important catalytic domains [13], and these were excluded from further consideration. Some slightly truncated proteins, such as CwatDRAFT_6607 and CwatDRAFT_1979, lacking structural motifs I, II or XI but possessing all catalytic motifs were retained. Nine proteins (including four STKs in marine unicellular *Prochlorococcus *and *Synechococcus*, 6803_slr1443, CwatDRAFT_0901, Tery_0088, Tery_4781 and Ava_3867) exhibit some deviations from the canonical motifs (Fig. 2) that were deemed tolerable. In all 286 putative STK sequences were considered in this study (Table 1).

The number of STK genes within the different cyanobacterial genomes varies considerably, from 0 to 56. Only one STK gene was found within the 5 fully sequenced *Prochlorococcus marinus *strains, in strain MIT9313. Eight out of nine sequenced *Synechococcus *strains contain STKs, with the marine *Synechococcus *strain WH8102 being the only exception. Three marine *Synechococcus *strains CC9902, CC9605, and CC9311 contain only one STK gene for each. Two *Synechococcus elongatus *strains PCC6301 and PCC7942, which are virtually identical in sequence, have five pairs of orthologous STK genes. More STK genes were found in the thermophilic *Synechococcus *strains JA-3-3Ab (8), JA-2-3Ba (8), and BP-1 (10). The other two unicellular cyanobacterial strains, *Gloeobacter violaceus *PCC7421 and *Crocosphaera watsonii *WH8501, have 14 and 28 STK genes respectively.

Filamentous diazotrophic cyanobacteria have the largest number of STK genes (40 for *Trichodesmium erythraeum *IMS101, 48 for *Anabaena *PCC7120, 53 for *Anabaena variabilis *ATCC 29413, and 56 for *Nostoc punctiforme *PCC73102). Among the unicellular cyanobacteria, the nitrogen-fixing unicellular strain *Crocosphaera *WH8501 has the biggest genome, the largest number of STKs and the highest percentage (0.52%) of STKs within protein-encoding genes (Fig. 1). STKs in *Prochlorococcus *MIT9313 represent only 0.04% of the total proteins, while in *Anabaena *ATCC29413, the percentage is twenty-fold higher at 0.85%. It is evident from these findings that filamentous diazotrophic cyanobacteria contain more STK genes than unicellular species (T-test, *p *< 0.001), and the number of STK genes is overrepresented in their genomes even after allowing for their larger genome sizes (Fig. 1).

### Conserved domain features

The core catalytic domain contains 12 conserved subdomains, within which a total of 12 amino acids are invariant or nearly invariant in eukaryotes: two G residues in subdomain I, K in subdomain II, E in subdomain III, D and N in subdomain VIb, D and G in subdomain VII, E in subdomain VIII, D and G in subdomain IX, and R in subdomain XI [13]. In cyanobacteria, 12 similar conserved subdomains were found and 10 residues are conserved well, with the exception of the two G residues in subdomain I, which are common but not invariant. It should be noted that additional conserved amino acids appear in cyanobacterial subdomains I, III, V, VIa, VIb, VII, VIII and IX (Fig. 2).

In eukaryotes, subdomain I serves as the ATP binding site (P-loop) and is characterized by the sequence GXGXXG. Cyanobacterial STKs are divergent in this motif with only 152 (53.3%) sequences exactly conserved in this domain, but 94.4% of them contain at least one Gly. It is possible that STKs lacking a conventional subdomain I may employ high energy phosphate compounds other than ATP, as does a PK from *Sulfolobus acidocaldarius *that was reported to use polyphosphate as phosphoryl donor [28,29]. Besides the conserved Gly, cyanobacterial subdomain I differs from its eukaryotic counterpart in that it starts at Tyr (93% of all cyanobacterial sequences) and ends with a non-polar residues (99%).

The Lys (96.1%) in subdomain II is important for maximal catalytic activity and helping anchor and orient ATP, and it is well conserved in cyanobacteria. But the upstream Ala described by Hanks and Hunter [13] is generally replaced by a different non-polar residue (97.2%). In subdomain III, Glu (86.0%) is not conserved well and is substituted by Gln in 27 sequences belonging to cyanobacterial Family III (cbSTKIII, details in section Structure and function). Upstream of the Glu, a conserved non-polar residue (99.3%) was found and most of them were Phe (87.7%).

Cyanobacterial subdomain V is much more conserved than the eukaryotic counterpart, which has no invariant residues. In contrast, the cyanobacterial subdomain possesses a highly conserved Gly (94.1%). This conserved residue may help anchor ATP by forming hydrogen bonds. Gly is also conserved in other bacteria (e.g. *Streptomyces coelicolor *[24]). In eukaryotes, subdomain VIa contributes to structural stabilization and possesses only a relatively conserved His. In cyanobacteria, besides this His (88.1%), a more conserved Leu (97.2%) has been found, which is also conserved well in *Mycobacterium tuberculosis *[23].

Subdomain VIb, a candidate for direct catalysis, is the most conserved motif in eukaryotes and is characterized as "hrDxkxxN". Cyanobacterial subdomain VIb is more conserved and is characterized as "HrDikPxN". His (100%) is the most conserved residue, followed by Asn (98.9%), Pro (98.6%), Asp (97.2%), Lys (86.7%), Ile (82.5%) and Arg (73.8%). Pro is also conserved in other bacteria, such as *Streptomyces coelicolor *[24] and *Mycobacterium tuberculosis *[23]. STKs are also referred as "RD" kinases because their activation requires the Arg and catalytic Asp in this subdomain. In cyanobacteria, however, Arg is not conserved well and it can be substituted by Lys, Gly, Cys or Gln. All members of cyanobacterial Family III (cbSTKIII) belong to the family of "KD" kinases. There are five cyanobacterial STKs lacking the highly conserved D, a candidate for the catalytic base. Among these five putative STKs, SpkE (Slr1443), which also lack of several other conserved residues, did not show any kinase activity [30]. Whether the other four could phosphorylate or not may need more experimental proofs.

Subdomain VII helps to orient the γ phosphate of ATP for transfer and is characterized by a conserved DFG triplet. The invariant Asp in subdomain VII, as well as the invariant Lys in subdomain II, was found to anchor and orient ATP [13]. In cyanobacteria, Asp (97.2%) and Phe (97.9%) are highly conserved but Gly (87.4%) less so. In 30 STK proteins from cyanobacterial Family III (cbSTKIII), Gly is substituted by Ser. The Lys residue often found in eukaryotes before the DFG triplet is not conserved in cyanobacteria, and the residues with near neutral polarity were replaced by non-polar ones (95.1%).

The typical subdomain VIII includes a highly conserved PE motif and plays a major role in the recognition of peptide or protein substrates. In cyanobacteria, the Pro residue (62.2%) of this motif is not well conserved, and the Glu residue (97.5%) is followed by a conserved Gln residue (90.5%) of unknown function. The PE motif in archaea, eubacteria and eukaryotes may represent an ancient and universal substrate recognition mechanism.

In subdomain IX, besides the Asp (99.3%), Gly (84.6%) and Pro (90.2%) residues conserved in eukaryotes, another three conserved amino acids were found: Thr (90.9%), Leu (87.0%) and Gly (80.1%). Gly is also conserved in *Mycobacterium tuberculosis *[23] and *Streptomyces coelicolor *[24].

In cyanobacteria there are six STKs, SpkA (Sll1574) [18], SpkB (Slr1697) [19], SpkC (Slr0599), SpkD (Sll0776), SpkF (Slr1225) [30], PknC (All4813) [31], which have been shown to phosphorylate themselves or other substrate proteins. From the sequence alignment it can seen that all six of these STKs have sequences that are in accordance with the conserved features found in cyanobacteria (Fig. 2).

### Structure and function

**Figure 3:**
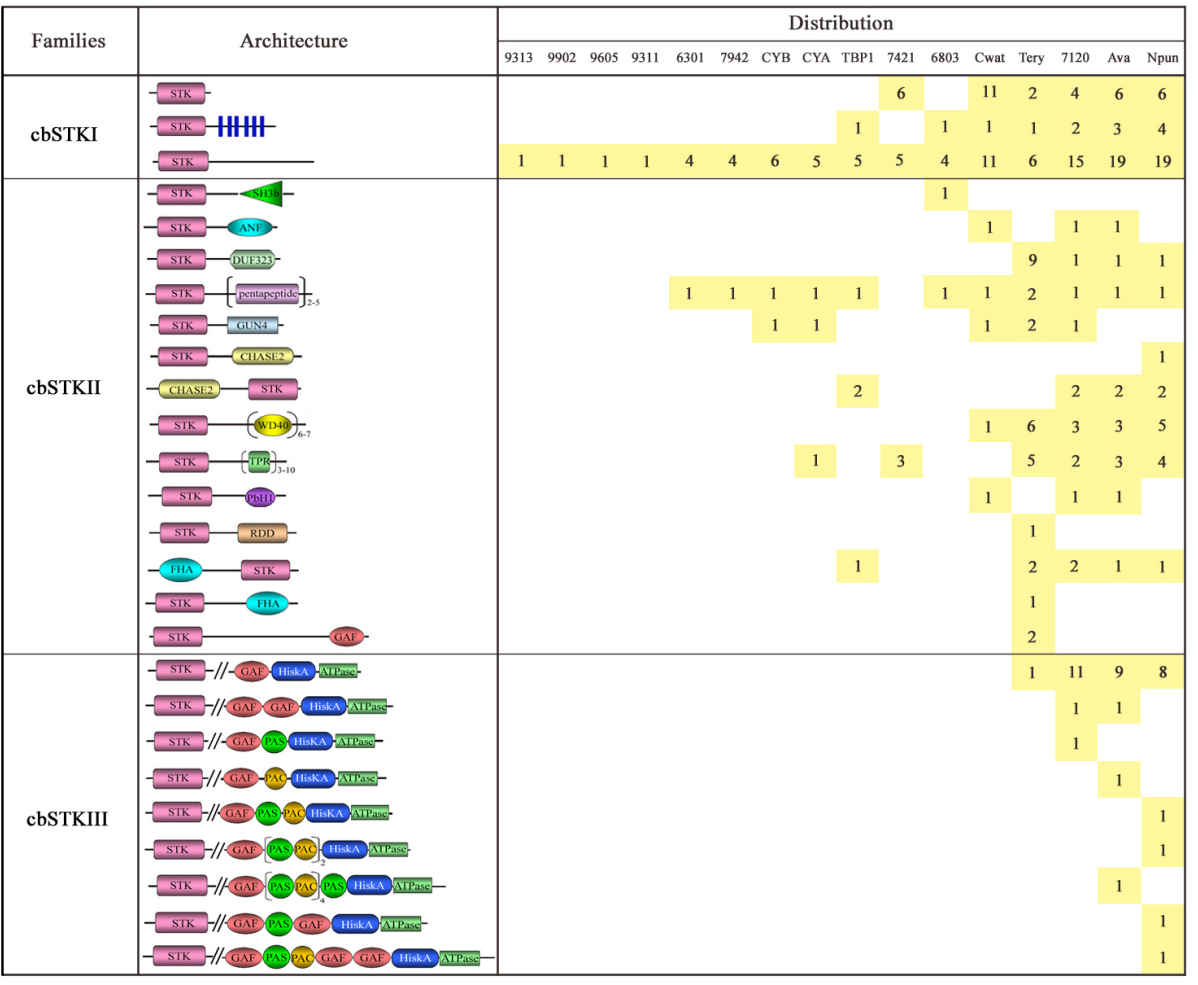
**Schematic representation and distribution of putative cyanobacterial serine/threonine protein kinases**. Strain names are as in Figure 1.

Cyanobacterial STKs were classified according to structural characteristics into three major families: cbSTKI, cbSTKII and cbSTKIII (Fig. 3).

Cyanobacterial STK Family I (cbSTKI) groups together the 155 (54.2%) STK proteins that possess no identifiable domain besides that associated with core catalysis. This family was further divided into three subfamilies. Subfamily I (cbSTKI-I) contains 35 proteins with fewer than 400 amino acids long and may be considered to possess the basic structure supplemented in other families by additional domains. Proteins in this family are found only in *Gloeobacter *PCC7421, *Crocosphaera *WH8501 and the four filamentous diazotrophic strains. Subfamily II (cbSTKI-TM) contains 13 proteins and is named for their 3 to 6 transmemberane (TM) domains behind the STK domains. These 13 proteins are distributed amongst unicellular *Synechocystis *PCC6803, *Thermosynechococcus elongatus *BP1, *Crocosphaera watsonii *WH 8501 and the four filamentous diazotrophic strains. Subfamily III (cbSTKI-other) consists of 107 STK proteins distributed amongst all 16 strains, each protein with more than 400 amino acids and possessing a long unidentified C-terminal domains. The functions of two cbSTKI proteins in *Synechocystis *PCC6803 and three in *Anabaena *PCC7120 have been confirmed by experiment. In *Synechocystis *PCC6803, Sll1574 (SpkA, I-other) was found to be involved in regulating cellular motility via phosphorylation of membrane proteins [18]. No phenotype defect could be discerned in a knockout mutant of Slr0599 (SpkC, I-other); however the protein was demonstrated to phosphorylate itself and some general substrate proteins [30]. In *Anabaena *PCC7120, Alr4366 (PknA, I-other) showed a complex pattern of regulation during heterocyst development [32], and Alr4368 (PknD, I-other) appeared essential for normal growth under diazotrophic conditions [20]. The function of Alr3732 (PknE, I-other) is known to be required for the formation of heterocyst envelope structures and nitrogen fixation [33].

Cyanobacterial STK Family II (cbSTKII) includes 92 (32.2%) proteins from 12 cyanobacterial species and are defined as those STK proteins that possess at least one additional domain in addition to their STK domains but do not possess Hik kinase domains. The additional domains with distinct functions are prevalent in protein kinases and phosphatases of prokaryotic and eukaryotic signal transduction systems. In total, 14 types of additional functional domains were identified in cyanobacterial STKs: PAS, PAC, GAF, ANF, WD40, FHA, GUN4, TPR, DUF323, PbH1, CHASE2, Pentapeptide, RDD, and SH3b. Most are involved in known signaling proteins and play various functions in the signal transduction process. For example, TPR (tetratricopeptide repeat), which are repeated in chaperone, cell-cycle, transcription, and protein transport complexes, form signaling domains in higher eukaryotes and may participate in the aggregation of proteins into multi-protein complexes [34]. GAF domains (cyclic GMP, adenylyl cyclases, FhlA) are linked to the binding of small-molecules, in particular the second messengers cyclic AMP (cAMP) and cGMP [35]. WD40 repeats, mainly in eukaryotic proteins, cover a wide variety of functions, for example, in adaptor/regulatory modules in signal transduction [36,37]. Finally, GUN4 is thought to participate in plastid-to-nucleus signaling by regulating magnesium-protoporphyrin IX synthesis or trafficking [38]. There are also some domains with unknown functions. These are found mainly in bacteria and include DUF323, Pentapeptide, and RDD. The putative functions of most of these additional domains have been described in detail by Krupa [25]. Most of these additional domains are located at C-terminal, with the exceptions of FHA and CHASE2.

Unicellular non-nitrogen-fixing strains have none or a limited number of additional domains. TPR is identified exclusively in *Gloeobacter violaceus *PCC7421, and DUF323 is the most prevalent in *Trichodesmium erythraeum *IMS101. *Crocosphaera watsonii *WH8501 has the lowest percent of additional domains except for marine unicellular non-nitrogen-fixing strains (Fig. 1). Filamentous diazotrophic cyanobacteria not only contain vast number of STKs but their STKs also recruit a diversity of additional domains. The STKs of *Anabaena *PCC7120, for example, contain 12 types of different additional domains. The marine filamentous diazotrophic strain *Trichodesmium *shows an exceptionally high percent (77.5%) and the greatest number of additional domains. Pentapeptide repeats, which have been found to participate in the accumulation of glycolipids into the heterocysts [39], are prevalent in both unicellular and filamentous cyanobacteria. WD40, containing 6 or 7 repeats, are found exclusively in nitrogen-fixing strains. Functions of three proteins in this family have been identified. Alr2502 (Pkn22, PbH1) was found to be associated with both iron-depletion and oxidative stress in *Anabaena *PCC7120 [40]. In *Synechocystis *PCC6803, Slr1697 (SpkB, Pentapeptide) was shown to be involved in the control of cell motility exclusive of positive phototaxis [19], and Sll0776 (SpkD, SH3b) could not be knocked out completely, indicating that it is essential for survival [30].

Kinases of STK Family III (cbSTKIII) are also named as dual protein kinases, as they contain both N-terminal STK domains and C-terminal His kinase domains, with at least one GAF domain in between. They are encountered in quite large numbers (38) in filamentous nitrogen-fixing strains, with the exception of *Trichodesmium erythraeum *IMS101, which possesses only one. This observation is consistent with an earlier comparative study reporting that GAF and PAS domains, possibly involved in signal recognition, are extremely abundant in *Anabaena*: 87 GAF domains in 62 ORFs and 140 PAS domains in 59 ORFs [41]. The expression of one cbSTKIII protein, Alr2258 (HstK) from *Anabaena *PCC7120, has been shown to depend on the type of available nitrogen source [42], and the expression of two others, Alr0709 (Pkn40) and Alr0710 (Pkn41), whose genes are adjacent along the chromosome and co-transcribed, are induced by iron deprivation and are under the control of the global nitrogen-regulator NtcA [43].

### Phylogenetic analysis

Phylogenetic analysis was performed using the conserved catalytic domains of STKs rather than their whole sequences, as the additional domains with their possibly separate evolutionary histories might confuse the phylogeny. The STK catalytic domains, about 280 amino acids in length, were identified using SMART and CDD databases [27,44].

**Figure 4:**
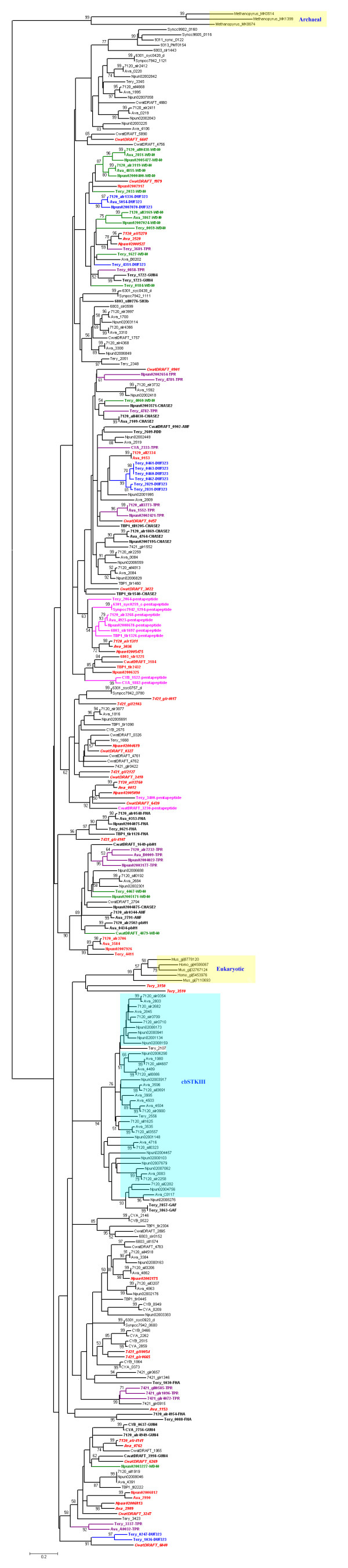
**Phylogenetic analysis of the conserved catalytic domains of STKs**. A phylogenetic tree based on the catalytic domains of cyanobacterial STKs was constructed as described in Methods. Strain names are as in Figure 1. Bootstrap values >50% are indicated on the branches. Additional domain names are also given following the gene names. STKs from subfamily cbSTKI-TM are marked in red and bold and those from cbSTKI-I are marked in red, bold, and italics. STKs with additional domains from cbSTKII are marked in bold and some major additional domains are marked in color: green WD40; blue DUF323; purple TPR; and magenta pentapeptide. Members of the family cbSTKIII family are highlighted in a cyan box. Archaeal and eukaryotic STK proteins are in yellow boxes.

The STK phylogenetic tree was rooted in the archaeal STKs, deviating from the canonical view of the universal phylogenetic tree rooted in eubacteria [45]. This STK phylogenetic tree indicates that these STK proteins display complicated relationships, at odds with the phylogeny of the species. STK proteins from the unicellular marine *Synechococcus *and *Prochlorococcus *cluster together and have a close relationship with Slr1443 from *Synechocystis *PCC6803. STKs from the unicellular cyanobacteria, *Synechocystis *PCC6803, *Synechococcus elongatus *PCC6301, *Synechococcus elongatus *PCC 7942, and *Thermosynechococcus elongatus *BP1 are dispersed throughout the phylogenetic tree. Seven pairs of STK orthologs are found in two *Synechococcus *strains, JA-2-3Ba and JA-3-3Ab, that share a close evolutionary relationship and are located at the bottom of 16S rRNA tree, along with *Gloeobacter violaceus *PCC7421 (Fig. 1). STKs from *Trichodesmium erythraeum *IMS101 and *Gloeobacter violaceus *PCC7421 each form several separate clusters indicating obvious lineage-specific duplication events in these strains. Most kinase sequences from *Anabaena *and *Nostoc *are orthologs for their close evolutionary relationships, while the nonorthologous STK genes in *Anabaena *and *Nostoc *may have been produced by recent gene duplication or lateral transfer. This phylogenetic tree shows also that cyanobacterial STKs do not cluster strictly according to their structural characteristics, except for members of cbSTKIII which are clustered together in a big clade. Most STKs of cbSTKII do not cluster according to their additional domains, while some with specific additional domains, such as Pentapeptide and WD40, do cluster together (Fig. 4).

**Figure 5:**
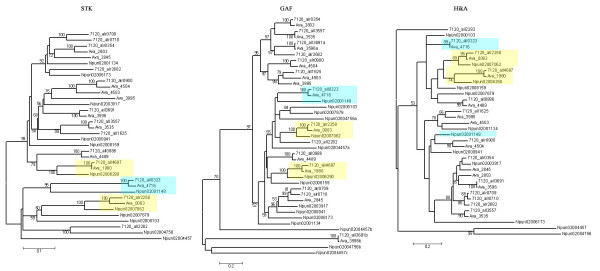
**Phylogenetic analysis of STK catalytic domains, GAF and HikA additional domains**. Phylogenetic trees were constructed from the amino acid sequences of the catalytic, GAF and HikA domains of dual protein kinases, as described in Methods. Branches with bootstrap support below 50% have been collapsed. Four proteins (*Anabaena PCC7120*_All3691, *A.variabilis*_3596, *N. punctiforme*_02004756 and *N. punctiforme*_02004457) have two GAF domains and one (*N. punctiforme*_02004457) has three. The strain abbreviations are as in Figure 1. Orthologous genes, conserved in three strains, were marked in yellow box. Members in cyan boxes are possible examples of domain shuffling.

Thirty-eight STKs in cbSTKIII belong to a large cluster that also includes four STK proteins that do not contain HisKA and ATPase domains and therefore have been placed in other families. Tery_2857 and Tery_3863 have GAF domains and belong to cbSTKII, while Tery_2107 and Npun02008276 have only the STK domains and belong to cbSTKI-other (Fig. 4). To test the evolutionary relationships amongst the catalytic domains of dual protein kinases, their HiskA domains, and their GAF domains, a detailed analysis was performed by constructing three separate phylogenetic trees (Fig. 5). Only two groups of orthologs as defined by the catalytic domain (highlighted in Fig. 5) clustered together in the other two phylogenetic trees, indicating that two original genes were formed and appeared in the lineage prior to the divergence of *Nostoc *and *Anabaena*. Genes ancestral to orthologs that are conserved only in *Anabaena *PCC7120 and *Anabaena *ATCC29413 are assumed to have formed before the divergence of these two strains. Apparent incongruities may be explained by new recruitment or domain shuffling. For example, the HiskA domain of *N. punctiforme *gene Npun02001148 appears to have a distinct history from the HiskA domains of *Anabaena *PCC7120 All0323 and *Anabaena *ATCC29413 Ava_4716, but the three proteins share a common history with respect to their catalytic and GAF domains (see proteins highlighted in blue in Fig. 5) The second GAF domains in All3691, Ava_3596, Npun02004457, and Npun02004756 and the third GAF domain in Npun02004457 bear only a distant relationships with other STK GAF domains in cyanobacteria, including those in the same genes, indicating a recently recruitment. Alr0709 and Alr0710 lie adjacent to each other on the chromosome of *Anabaena *PCC7120 and show 75% similarities, indicating a recently gene duplication.

## Discussion

Ser/Thr protein kinases, members of signal transduction systems, play important roles in responses to the environmental changes and intra-cellular signals [29]. This may be vital for their host cyanobacteria, which originated 2.5–3.5 billion years ago and exhibit the widest range of diversity in growth habitats of all photosynthetic organisms. Some of these kinases have demonstrated, in at least one case, possibly essential roles, illustrated by Sll0776 (SpkD) in *Synechocystis *PCC6803 which could not be completely knocked out [30].

The STK proteins used in this study were identified by Local Blast rather than from the COG database in IMG, and were manually checked to avoid false-negative and false-positive hits that commonly arise during large-scale automated analyses. Sequence alignment of conserved catalytic domains show that cyanobacterial STKs contain nearly the same subdomains as eukaryotic STKs but seem to be more conserved except in subdomain I. Eukaryotic STK proteins shared 12 nearly identical residues. Whereas a total of 15 highly conserved (shared by >90% sequences) sites were found in cyanobacteria, seven of them were the same in eukaryotes. Such conserved motifs and amino acids may provide useful targets to evaluate their functions, and the high proportion of conserved residues shared by both eukaryotic organisms and cyanobacteria indicate a quite similar mechanism for domain organization and catalyzation.

Signal transduction systems provide basic mechanisms for cellular responses to environmental changes and connect environmental signals to cellular activities [29]. Thus different distributions of STKs through gene gain or loss may reflect various environmental selective pressures. Such as *Synechococcus *strains, which have similar genome size, hold different numbers of STK genes from zero to ten mainly for the different environmental conditions. Moreover, the number of STK genes in cyanobacteria is a function of the genome size, ecophysiology, and physiological properties of organism, as has been noted in general for bacteria [46,47]. STK genes are not ubiquitous in cyanobacteria. They are absent in four *Prochlorococcus *strains and one marine *Synechococcus *WH8102. Previous study showed that signal transduction and environmental stress response systems are dramatically reduced in marine unicellular *Prochlorococcus *and *Synechococcus *[48], both of whom live in the oligotrophic open ocean, and the major driving force behind might be a selective process favoring the adaptation of these cyanobacteria to adapt to the oligotrophic environment [49]. In contrast, filamentous heterocystous cyanobacteria, which differentiate heterocysts in response to the absence of combined nitrogen, and which display physiological and ecological properties including broad symbiotic competence with plants and fungi [41], showed a disproportionate number of STK genes.

Although Ser/Thr protein kinases were first discovered in eukaryotes and are evidently more widely dispersed amongst them, this is an insufficient basis to conclude that STKs originated in eukaryotes and were obtained by prokaryotes through lateral gene transfer. Zhang [50] and Leonard [51] have demonstrated that an ancestral protein kinase existed prior to the divergence of eucarya, bacteria, and archaea. The phylogenetic tree from our analysis shows STKs from archaea clustering at the root and cyanobacterial STKs clustering together with those from eukaryotes. The phylogenetic tree of cyanobacterial STKs, which was constructed by their conserved catalytic domains, is very complicated, and the distribution of STKs along the tree does not follow their domain structures or species phylogenies, indicating that frequent gene gain-and-loss events in cyanobacteria may obscure the actual relationships of STKs.

Besides gene gain-and-loss, novel proteins could also be generated by inserting or shuffling their domains. In these 21 cyanobacteria genomes, a total of 14 types of additional functional domains were found, much more than in other bacteria such as *Mycobacterium *and *Streptomyces *[23,24]. Additional domains are usually employed as sensor response modules [52-55] and can help organisms to assemble a sophisticated signal transduction apparatus. Their modularity may facilitate their spread into new genes and the rapid creation of novel functions [17]. Some additional domains (e.g. TPR) may have existed in the putative common ancestor of all life forms, and some (e.g. FHA) are thought to have been transferred from eukaryotes to bacteria [37]. Some prevalent additional domains, such as pentapeptides that are widely distributed in bacteria, may have been obtained from the ancestor at an early evolutional stage. The species-specific additional domains might have been recruited after speciation and duplicated recently, as in the example of the DUF323 domain in *Trichodesmium erythraeum *IMS101. Four STKs with DUF323 domains of *T. erythraeum*_0640–0643, which have high sequence similarities and neighbor on the chromosome, are closest paralogues and might have arisen from a recent duplication. STKs that cluster together according to their conserved catalytic domains do not always do so according to their additional domains, indicating the lateral recruitment of additional domains by STKs. A similar phenomenon was previously observed regarding the GAF and PAS domains found in PPM-family protein phosphatases in *Streptomyces *[56]. The two clusters with FHA and Pentapeptide domains are indicative of an early recruitment. GAF domains are prevalent in cyanobacterial two-component system and exist in all sequenced cyanobacterial genomes. But different STK orthologues did not recruit the same GAF and HiskA orthologs. It is assumed that HiskA and GAF domains were added to STK domains together and followed by domain shuffling. Orthologs that are conserved in three strains indicate their existence before the divergence of *Anabaena *and *Nostoc*, and the orthologs conserved in only two *Anabaena *strains suggest that they appeared before the divergence of the two *Anabaena *strains and after the split of *Anabaena *and *Nostoc*. Some sequences appear to have lost the additional domains, and some appear to have gained PAC and PAS domains, indicating that gene shuffling, deletion and insertion, as well as gene duplication, occurred during evolution.

Results from PPM-family protein phosphatases in *Streptomyces*, showed that PAS and GAF domains were clustered by physiological functions rather than taxonomic relationship [56]. So functions of substrate recognition in cyanobacterial STKs may be conferred largely by their additional domains too. For example, the CHASE (cyclase/histidine kinase-associated sensing extracellular) domain, always followed by three transmembrane regions, is present in more than one type of sensory proteins and can recognize several signals, such as cytokines and short peptides that are important for the development of an organism [57]. STKs, lacking transmembrane regions and acting as a receptor-like protein kinase in the cytoplasm, also appeared to have recruitted some additional domains such as FHA and GAF. The FHA domain is thought to play roles in a wide range of processes, including intracellular signal transduction, transcription, protein transport, DNA repair, and protein degradation [12]. The GAF domain family, linked to the binding of second messengers, is involved in cyclic nucleotide signaling, transcription, phototransduction, and probably many more unidentified processes [58]. Therefore, besides the conserved catalytic domain, the additional domains also play important roles in functions of kinases.

## Conclusion

The availability of genome sequences provides a good opportunity for comparative analysis of gene families. Ser/Thr protein kinases have important effect on cyanobacteria living, as well as two component system. A total of 286 putative STK genes have been identified from 21 species of cyanobacteria using BlastP, TblastN and ClustalW, and 19 were not annotated originally as STKs. The number of STKs varied as a function of the genome size, ecophysiology and physiological properties. Fourteen types and 131 additional domains embedded in STK genes may assist their signal recognition functions. A similar catalytic mechanism was inherited with conventional conserved motifs, amino acids, and some uniquely cyanobacterial conserved residues. Gene duplication, loss, shuffling, insertion, and/or horizontal transfer appear to have played important roles during the evolution of cyanobacterial STKs.

## Methods

Twenty-one species of cyanobacteria, including *Prochlorococcus, Synechococcus, Synechocystis, Crocosphaera, Gloeobacter, Trichodesmium, Anabaena *and *Nostoc *were used in this analysis. Since sequences of three *Synechococcus *strains RS9917, WH5701, WH7805 and one *Prochlorococcus marinus *strain MIT9312 are not yet fully released, they were not considered in our comparisons. All 21 genome sequences (as of Jan 2007) were accessed from IMG [59] in FASTA format. Four archaeal STK proteins in *Methanopyrus kandleri *AV19 were downloaded from the IMG database, and five eukaryotic STK proteins from human and mouse were downloaded from NCBI [60].

In order to identify genes that may encode STKs, a set of proven cyanobacterial STKs was used to search individual cyanobacterial genomes. The proven cyanobacterial STKs used were alr2502 (Pkn22) [40], alr4366 (PknA) [32], all4813 (PknC) [31], alr4368 (PknD) [20], alr3732 (PknE) [33] from *Anabaena *PCC 7120, and sll1574 (SpkA) [18], slr1697 (SpkB) [19], slr0599 (SpkC) [30], sll0776 (SpkD) [30] from *Synechocystis *PCC6803. Sll1574 (SpkA) is interrupted by an insertion element in the sequenced strain, and in this paper "Sll1574" refers to the reconstituted Sll1574-1575. BlastP and TblastN were conducted locally to search all proteins from each of the 21 cyanobacteria, using a threshold e-value of 1e-10. Proteins found by this method that fit the criteria for a genuine STK (see below) were added to the query set for another round of Blast searches. The procedure was continued until no new proteins were found.

Proteins identified by Blast were aligned by using ClustalW program [58] with a gap opening penalty of 10, a gap extension penalty of 0.2, and Gonnet as the weight matrix. Alignment of query sets were examined by inspection of apparent Motifs I through XI per Hanks and Hunter [13] (Figure 2). A protein was accepted as an STK if it was possible to recognize the most conserved subdomains (i.e. II, V, VIb, VII, VIII, IX, and XI) and if those conserved amino acid residues known to participate in the function of STKs were present. Some minor deviations were tolerated, considered on a case-by-case basis. The ultimate decision was in the end subjective but in most cases quite clear cut.

Structure analyses of the obtained STKs were performed using the SMART [27,61] and CDD (Conserved Domains Database) [44,62] databases, relying on hidden Markov models and Reverse Position-Specific BLAST separately. Sequences of the catalytic domains (about 280 aa in length) and additional domains used for phylogenetic tree construction were obtained from the SMART database and sequences with poor instances of Motif I and XI were checked manually. Trees based on 16s rRNA, STK conserved catalytic domains, GAF domains (mean of 150–200 aa), and HiskA domains (mean of 65–100 aa) were constructed using NJ methods of the MEGA (Version 3.0) package [63], and the reliability of each branch was tested by 1000 bootstrap replications.

## Authors' contributions

XWZ, FQZ and SQ devised the overall strategy for these studies. XWZ performed all database searches, acquired the sequence data, and prepared all figures and tables. XWZ and FQZ performed the conserved domain, structure, alignments, and phylogenetic analyses. XWZ, FQZ, YY, CWL and XYG jointly wrote the paper, and all authors have read and accepted the final version of the manuscript.
